# Reproduction of a Canine Tooth, at Thirty-Nine Years of Age

**Published:** 1860-01

**Authors:** G. F. J. Colburn

**Affiliations:** Newark, N. J.


### ARTICLE X.

### *Reproduction of a Canine Tooth, at thirty-nine years of age.*

MESSRS EDITORS:

I have just seen a remarkable case of reproduction in the mouth of a lady wearing a set of ten upper teeth, on gold plate. Some fifteen months ago, in preparing her mouth, I had considerable difficulty in removing the eye tooth root on the right side, it being so decayed as to repeatedly break under the operation. To the end of the fang was attached a large ulcer. Some six months after, when the patient came to have the impression taken, she complained of a "sore lump," that had appeared where the eye tooth root had been removed. On examination, sure enough, there was a protuberance of similar form and texture, as that caused by a tooth, endeavoring to break through its fleshy covering. But as the patient had passed the years of childhood, and arrived at that age when new teeth are scarce, unless supplied by the dentist, the idea of cutting a new tooth where one had but a short time before been removed, never entered the head of operator or patient. The lump was supposed to originate from some irritation in eating. The impression was taken, the teeth inserted, and not until this morning, more than a year after, was anything heard of the patient, who would not have called at this time if it "had not been for the breaking" of a tooth which she wished replaced. While speaking of the plate, she remarked, that it was very loose, and did not fit her mouth, as when made, which she thought was owing to a new tooth she was cutting. I laughed at the idea, of a woman of 40 years old, getting a new tooth, but on examining the mouth, sure enough, where I had such difficulty in removing the old tooth, and where the lump had been, was the enameled surface of what appeared to be another; instead of the cutting edge projecting, as in the usual way, the anterior surface of the crown presented itself parallel with the gum, occupying the place of the lateral incisor. After much persuasion, I removed a perfectly formed canine tooth, and of the usual size. The lady departed, expressing herself very grateful that the "pesky" thing was out of the way, believing that she should after this be "*cuter*" than ever, having cut her eye teeth the third time.

Yours,

G. F. J. COLBURN, D. D. S.

NEWARK, N. J., *Nov.* 1*st*, 1859.

